# Outcomes of kidney transplantation in Alport syndrome compared with other forms of renal disease

**DOI:** 10.1080/0886022X.2016.1262266

**Published:** 2016-12-05

**Authors:** Yvelynne P. Kelly, Anish Patil, Luke Wallis, Susan Murray, Saumitra Kant, Mohammed A. Kaballo, Liam Casserly, Brendan Doyle, Anthony Dorman, Patrick O’Kelly, Peter J. Conlon

**Affiliations:** a Department of Nephrology, Beaumont Hospital, Dublin, Ireland;; b Royal College of Surgeons in Ireland, Dublin, Ireland;; c Department of Nephrology, University Hospital Galway, Galway, Ireland;; d Department of Nephrology, Cork University Hospital, Cork, Ireland;; e Department of Nephrology, University Hospital Limerick, Dooradoyle, Limerick, Ireland;; f Department of Pathology, Beaumont Hospital, Dublin, Ireland

**Keywords:** Kidney transplantation, transplant outcomes, transplant rejection, transplant complications, hereditary nephritis, hematuria

## Abstract

**Introduction:** Alport syndrome is an inherited renal disease characterized by hematuria, renal failure, hearing loss and a lamellated glomerular basement membrane. Patients with Alport syndrome who undergo renal transplantation have been shown to have patient and graft survival rates similar to or better than those of patients with other renal diseases.

**Methods:** In this national case series, based in Beaumont Hospital Dublin, we studied the cohort of patients who underwent renal transplantation over the past 33 years, recorded prospectively in the Irish Renal Transplant Registry, and categorized them according to the presence or absence of Alport syndrome. The main outcomes assessed were patient and renal allograft survival.

**Results:** Fifty-one patients diagnosed with Alport syndrome in Beaumont Hospital received 62 transplants between 1982 and 2014. The comparison group of non-Alport patients comprised 3430 patients for 3865 transplants. Twenty-year Alport patient survival rate was 70.2%, compared to 44.8% for patients with other renal diseases (*p* = .01). Factors associated with patient survival included younger age at transplantation as well as differences in recipient sex, donor age, cold ischemia time, and episodes of acute rejection. Twenty-year graft survival was 46.8% for patients with Alport syndrome compared to 30.2% for those with non-Alport disease (*p* = .11).

**Conclusions:** Adjusting for baseline differences between the groups, patients with end-stage kidney disease (ESKD) due to Alport syndrome have similar patient and graft survival to those with other causes of ESKD. This indicates that early diagnosis and management can lead to favorable outcomes for this patient cohort.

## Introduction

Alport syndrome is an inherited renal disease, first described in 1927^1^ which is characterized by hematuria, renal failure, hearing loss, lenticonus, retinal flecks,^2^ a lamellated glomerular basement membrane^3^ and mutations in the *COL4A5* or *COL4A3/COL4A4* genes^4^ leading to abnormal type-IV collagen composition.^5^ The prevalence of the disease is estimated at 1 in 50,000 live births.^6^ Eighty-five percent of families have X-linked inheritance with mutations in *COL4A5.*^7^ Most of the others have autosomal recessive inheritance, with homozygous or compound heterozygous mutations in both gene copies of *COL4A3* or COL4A4.^8^ Autosomal dominant inheritance is rare and results from heterozygous *COL4A3* or *COL4A4* variants.^9^

Patients with X-linked Alport syndrome who undergo renal transplantation have been shown to have patient and graft survival rates similar to or better than those of patients with other renal diseases.^10^ In a recent publication from the ANZDATA registry survival post 1st renal transplantation for Alport patients was found to be superior to that of patients with other renal diseases in an earlier time period (1965–1995), whereas survival for Alport patients post-transplantation was comparable to that of non-Alport patients in the more modern time era (1996–2010). The authors found that younger age at transplantation and more recent transplant era were the only independent predictors of renal transplant survival after adjusting for donor source, age, gender, race, and time from dialysis to renal transplant.^11^ It has been reported that 3–5% of males develop *de novo* anti-GBM disease with rapid allograft loss after transplantation. This phenomenon may manifest immediately after transplantation or later, though usually within the first 1 year. The etiology of *de novo* anti-GBM disease is thought to be due to the presence in the transplanted kidney of antigenic epitopes that are lacking in the native kidneys of Alport patients.^12^

## Methods

In this large national case series, based in Beaumont Hospital, Dublin, Ireland, we studied the cohort of patients who underwent renal transplantation there over the past 33 years, recorded prospectively in the Irish Renal Transplant Registry, and categorized them according to the presence or absence of Alport syndrome. The main outcomes that were assessed were patient and renal allograft survival. Patients with Alport syndrome were identified using a database search of the Irish Renal Transplant Registry and their diagnosis was confirmed using histopathological and online clinical records.

Patients who had received a simultaneous kidney-pancreas transplant were excluded from the statistical analysis. The immunosuppression regime utilized for all patients transplanted in Beaumont Hospital has been described in previous publications.^13^ Comparisons between the two groups were made for continuous and categorical variables. For the former, either mean and standard deviation (SD) or median and interquartile ranges (IQR) were used with the appropriate statistical tests (either *t*-tests or Mann–Whitney U-tests). Pearson Chi-squared tests were used to assess the difference between groups for the latter variables. Multivariable Cox proportional hazards models were performed for the principal outcomes with clinical and patient variables to allow for potential confounding with the main predictor variable.

Statistical analysis was performed using STATA (Version 13, College Station, TX) software. Results for all statistical tests were deemed significant for *p* values less than 0.05.

The RCSI teaching hospitals ethical approval committee granted permission for use of records from the Irish Renal Transplant Registry Database, maintained in Beaumont Hospital Dublin Ireland.

## Results

A total of 3918 kidney transplants were performed in 3481 patients in Beaumont Hospital between the years 1982–2014. Sixty-two grafts (1.6%) of these kidney-only transplants were performed in 51 patients with end-stage kidney disease (ESKD) caused by Alport syndrome. The baseline and transplant characteristics of patients with Alport and non-Alport renal disease are displayed in Table 1. The mean age at ESKD was 31 years for patients with Alport syndrome compared to 40 years for those with other forms of renal disease (*p* < .001). There were proportionally more males in the Alport cohort and more recipients of living donor transplants and they had lower levels of PRA (Table 1).

**Table 1.** Baseline and transplant characteristics of patients with Alport syndrome compared to patients with end-stage kidney disease (ESKD) from other causes.

Variables	Non-Alport renal disease *N* = 3856	Alport syndrome *N* = 62	*p* values
Age at transplant, mean (sd)	41.9 (16.3)	33.31 (12.65)	<0.001
Age at ESKD, mean (sd)	39.7 (16.0)	30.7 (12.3)	<0.001
Sex, % male/female	63.6/36.4	83.9/16.1	0.001
Time on transplant waiting pool in months, median (IQR)	13.1 (6.2–24.6)	12.9 (4.4–22.4)	0.515
Cold ischemic time in hours, median (IQR)	19 (16–22)	20 (17–23)	0.232
HLA mismatches, median (IQR)	3 (2–4)	3 (2–4)	0.372
Transplant number, % first transplants	86.0	80.6	0.232
Living donor transplants, %	7.4	14.5	0.034
Pre-emptive transplants, %	6.0	1.6	0.146
PRA groups,% 0–10, 11–49, 50–100	62.8/18.0/19.2	76.7/6.7/16.7	0.045

**Figure 1.**
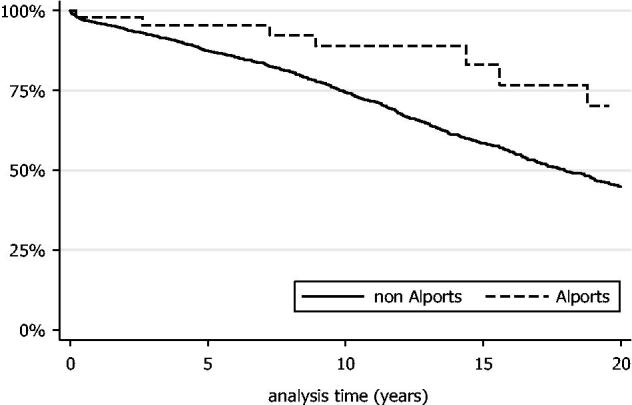
Kaplan–Meier curve of patient survival within the Alport cohort compared to patients with other forms of renal disease.

Twenty-year Alport patient survival rate was 70.2% (95% CI: 44.5–85.7%), while the 20-year survival rate for patients with other renal diseases was 44.8% (95% CI: 41.9–47.7) (*p*= .01; Figure 1). A Cox proportional hazards multivariable model demonstrated younger age at transplantation and female sex, younger donor age, shorter cold ischemia time and fewer episodes of acute rejection as being significantly associated with an increased patient survival (Table 2). No patients developed *de novo* anti-GBM disease postrenal transplantation.

**Table 2.** Multivariable model of risk factors for patient survival between the Alport and non-Alport group.

Variables	Hazard ratio	95% Confidence interval	*p*>|*z*|
Alport syndrome	0.72	0.32–1.61	0.42
Recipient age at transplant	1.06	1.05–1.06	<0.01
Male recipient	1.29	1.11–1.51	<0.01
Donor age	1.00	1.00–1.01	0.04
Male donor	0.96	0.83–1.11	0.62
HLA mismatch	0.95	0.90–1.01	0.09
Cold ischemic time	1.02	1.01–1.04	<0.01
PRA group	1.08	0.96–1.20	0.19
Delayed graft function	1.12	0.93–1.37	0.24
Acute rejection	1.41	1.21–1.65	<0.01

**Figure 2.**
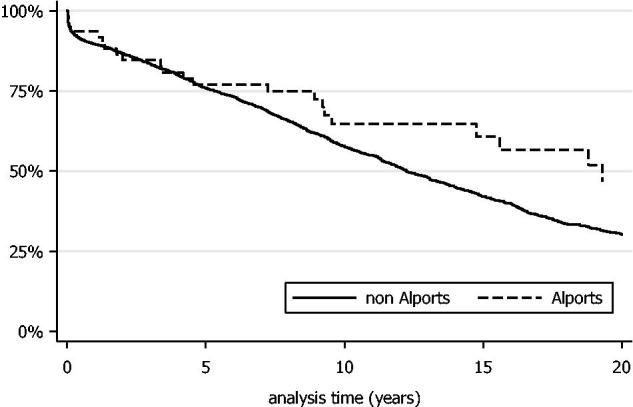
Kaplan–Meier curve of graft survival within the Alport cohort compared to patients with other forms of renal disease.

Median transplant survival for Alport patients was 19.3 years (95% CI: 9.5–24.9 years) compared to 12.2 years for non-Alport patients (95% CI: 11.8–13.0 years) with no significant difference in graft survival detected between the two groups, (*p* = .11). Twenty-year graft survival was 46.8% (95% CI: 28.2–63.3%) for patients with Alport syndrome compared to 30.2% (95% CI: 28.0–32.5%) for patients with other renal diseases (Figure 2). A Cox proportional hazards model demonstrated that older age at transplantation, older donor age, female donor, increased number of HLA mismatches, longer cold ischemic time and episodes of acute rejection were significant risk factors for graft loss (Table 3).

**Table 3.** Multivariable model of risk factors for graft survival between the Alport and the non-Alport group.

Variables	Hazard ratio	95% Confidence interval	*p* values|
Alport syndrome	0.73	0.46–1.15	0.17
Recipient age at transplant	1.01	1.01–1.02	<0.01
Male recipient	1.12	1.00–1.25	0.05
Donor age	1.00	1.00–1.01	0.01
Male donor	0.87	0.78–0.97	0.01
HLA mismatch	0.96	0.92–0.99	0.03
CIT	1.02	1.01–1.03	<0.01
PRA group	1.03	0.95–1.12	0.42
Delayed graft function	1.12	0.96–1.29	0.15
Acute rejection	1.60	1.43–1.79	<0.01
Previous transplant	1.06	0.9–1.24	0.50

## Discussion

Our study of the transplant-related outcomes of Alport syndrome is one of the largest of its kind and followed a national cohort of patients over a substantial 33-year time period.

This national case series of 3918 kidney-only transplants in Ireland between 1982–2014 showed a low prevalence of Alport syndrome within the cohort (62 grafts; 1.6%). The majority of patients with Alport syndrome who progressed to ESKD were however successfully transplanted with good transplant survival times. Twenty-year patient survival with Alport syndrome was significantly greater than that of patients with other renal diseases. This was accounted for mainly by younger age at transplantation as well as differences in gender distribution, donor age, cold ischemic time, and acute rejection episodes. A similar survival advantage was noted by Mallett et al.^11^ and a matched European registry cohort study.^14^ Mallett et al. also found an allograft survival benefit for patients with Alport syndrome treated in an earlier era (1965–1995). Allograft survival was however similar for their cohort for both Alport and non-Alport ESKD patients in the modern era (1996–2010).

In our case series, there was no significant difference in graft survival time between patients with Alport syndrome and those with other renal diseases, taken from the Irish Transplant Registry; however the long-term graft survival demonstrated was excellent for both Alport and non-Alport allograft recipients. Temme et al.^14^, however, found that patient and transplant survival in the Alport cohort in the ERA-EDTA registry was better than that of age-matched controls in their prospective study of dialysis patients from 14 national European registries between 1990–2009. The authors hypothesized that this was due to the absence of other vital organ involvement in Alport syndrome, and the lack of recurrence post transplantation compared to other systemic inflammatory causes of ESKD. There were several methodological differences between this study and our patient cohort, such as the exclusion of female cases from the final analysis. The much larger pan-European case numbers allowed the authors to perform much closer case-control matching, which may have allowed for a survival advantage for Alport patients to be shown more clearly, even when adjusted for age.

We reviewed the immunofluorescence staining for all native and transplant biopsies performed for the Alport cohort and no evidence of *de novo* anti-GBM disease was found. This may suggest that this epitope-mediated phenomenon may occur more rarely than was previously thought.

## Conclusions

Alport syndrome is a rare inherited renal disease. In this large national cohort study spanning 33 years, we have described the transplant-related outcomes of a large number of patients with Alport syndrome compared with other forms of renal disease. The majority of patients with Alport syndrome who progressed to ESKD were transplanted with good long-term outcomes. No patients developed *de novo* antiglomerular basement membrane disease post-transplantation in our cohort. Adjusting for baseline differences between the groups, patients with ESKD due to Alport syndrome have similar patient and graft survival to those with other causes of ESKD. This lends credence to the premise that early disease diagnosis and careful management can lead to favorable long-term outcomes for this patient group. In terms of future directions, we plan to genotype our national cohort to examine patterns of Alport disease inheritance in Ireland in order to assess the utility of genetic diagnosis for this disease on long-term prognostication and management of kindreds with hereditary nephritis within an Irish context.
